# Tuberculoid Hansen’s – a case report

**DOI:** 10.1186/1471-2334-12-S1-P36

**Published:** 2012-05-04

**Authors:** Jaisri Anantha Padmanaban

**Affiliations:** 1 Southern Railway Headquarters Hospital, Perambur, Chennai, India

## Background

Leprosy also known as Hansen’s disease is still prevalent. This case is presented to highlight the fact that it can occur even in immunocompetent individuals in the form of Tuberculoid Hansen’s and for the fact that a single bacillus was identified on tissue sections stained by the Fite-ferako technique, which is rare in tuberculoid Hansen’s.

## Case report

A fifty-three year old male patient presented with a hypopigmented patch over the left forearm measuring four centimeters for the past one month. He had numbness also over the patch. A skin biopsy was taken from the patch and sent for histopathological examination.

## Histopathology

Sections showed skin with the subepidermal region showing numerous epithelioid granulomas surrounded by lymphocytes. Stain with Fite-ferako’s technique was positive with a single bacillus within one epithelioid cell. A diagnosis of tuberculoid Hansen’s was made. The patient responded very well with rifampicin, dapsone and clofazimine treatment.

## Discussion

Tuberculoid Hansen’s is a disease occurring in an immunocompetent individual with granulomatous reaction in the tissue. Usually no bacillus is identifiable. Nerve involvement is common with thickening of the nerve and numbness of the corresponding region. Leprosy caused by *Mycobacterium leprae* is one of the oldest diseases on earth. It has its earliest description in India and China as early as 600 BC. Several clinical classifications are available. The Ridley-jopling classification correlates clinical findings with histopathology. Hansen’s is either paucibacillary (indeterminate, tuberculoid and borderline tuberculoid) and multibacillary (borderline borderline, borderline lepromatous and lepromatous lepromatous).

